# Intraspecific variation of a dominant grass and local adaptation in reciprocal garden communities along a US Great Plains’ precipitation gradient: implications for grassland restoration with climate change

**DOI:** 10.1111/eva.12281

**Published:** 2015-07-16

**Authors:** Loretta C Johnson, Jacob T Olsen, Hannah Tetreault, Angel DeLaCruz, Johnny Bryant, Theodore J Morgan, Mary Knapp, Nora M Bello, Sara G Baer, Brian R Maricle

**Affiliations:** 1 Biology, Kansas State UniversityManhattan, KS, USA; 2 Department of Biological Sciences, Fort Hays State UniversityHays, KS, USA; 3 Department of Agronomy, Kansas State UniversityManhattan, KS, USA; 4 Statistics, Kansas State UniversityManhattan, KS, USA; 5 Plant Biology and Center for Ecology, Southern Illinois UniversityCarbondale, IL, USA

**Keywords:** abiotic stress, *Andropogon gerardii*, competition, drought, ecotypic variation, grassland restoration, Great Plains grasslands, local adaptation, reciprocal gardens

## Abstract

Identifying suitable genetic stock for restoration often employs a ‘best guess’ approach. Without adaptive variation studies, restoration may be misguided. We test the extent to which climate in central US grasslands exerts selection pressure on a foundation grass big bluestem (*Andropogon gerardii*), widely used in restorations, and resulting in local adaptation. We seeded three regional ecotypes of *A. gerardii* in reciprocal transplant garden communities across 1150 km precipitation gradient. We measured ecological responses over several timescales (instantaneous gas exchange, medium-term chlorophyll absorbance, and long-term responses of establishment and cover) in response to climate and biotic factors and tested if ecotypes could expand range. The ecotype from the driest region exhibited greatest cover under low rainfall, suggesting local adaptation under abiotic stress. Unexpectedly, no evidence for cover differences between ecotypes exists at mesic sites where establishment and cover of all ecotypes were low, perhaps due to strong biotic pressures. Expression of adaptive differences is strongly environment specific. Given observed adaptive variation, the most conservative restoration strategy would be to plant the local ecotype, especially in drier locations. With superior performance of the most xeric ecotype under dry conditions and predicted drought, this ecotype may migrate eastward, naturally or with assistance in restorations.

## Introduction

Worldwide, temperate grasslands are one of the most threatened ecosystems (Hoekstra et al. 2005). Forty-five percent of temperate grassland and savanna biome area has been converted, primarily for agricultural purposes, with the second highest conversion after rainforest (Hoekstra et al. 2005). Of all biomes, only 4.8% of the grassland biome area is protected, the least of any biome, even more threatened than tropical forests (Hoekstra et al. 2005). Within the US grasslands, only 4% of prairie remains (Samson and Knopf 1994; Samson et al. 2004), with the greatest extent in the Flint Hills of Kansas and Nebraska. Small areas of prairie remain in the fertile mesic regions of the eastern Great Plains, where ∼99% has been converted to row crop agriculture. To exacerbate conservation threats from land conversion, grasslands are among the hottest spots for species diversity (WRI 2000). Thus, much conservation interest exists in restoration and identifying best restoration practices to establish, maintain, and enhance diversity (Montalvo et al. 1997; Packard and Mutel 1997; Rice and Emery 2003; Maschinski et al. 2013).

Identifying suitable genetic sources for restoration usually employ a ‘best guess’ approach (Broadhurst et al. 2008), often planting the ‘local ecotype’. This restoration strategy is common especially when key adaptive variation studies (reciprocal garden studies) are lacking.

During restoration of prairies in the eastern Great Plains, where most of the prairie had been converted to row crop agriculture, seeds are often sourced from the few remaining small, fragmented, and potentially genetically eroded (Bijlsma and Loeschcke 2012) local populations. While conservation plantings on marginal lands in the Conservation Reserve Program throughout the US Great Plains alone cover 4.3 million ha in a five-state region (http://www.fsa.usda.gov/programs-and-services/conservation-programs/reports-and-statistics/conservation-reserve-program-statistics/index), these restoration efforts have often used genetic cultivars (Soil Conservation Sciences 1990; Baer et al. 2014), another ‘best guess’ approach. These cultivars have often been intensively selected rather than using seed sourced from natural prairie populations or local ecotypes (McKay et al. 2005). Without adaptive variation studies (Falk et al. 2006) as presented here, restoration efforts may be misguided.

In spite of the rapid pace of grassland restoration, much fundamental knowledge is still lacking regarding adaptive variation and local adaptation in grassland species. In fact, few researchers have performed empirical measures of adaptive potential of a species within grasslands. One of the most comprehensive grassland ecotype studies to address this was published in the middle of the last century (McMillan 1959, 1965). Clearly, with the pace of environmental change and restoration, it becomes imperative to expand our knowledge on the extent of local adaptation in dominant grasses that often serve as foundation species in restorations (Baer et al. 2002, 2014). Knowledge of present within-species diversity and adaptive response, especially in changing climates and in increasingly fragmented populations, becomes critical to inform restoration efforts (Harris et al. 2006).

This study focuses on big bluestem *(Andropogon gerardii* Vitman), a dominant C_4_, long-lived, perennial outcrossing grass (Weaver and Fitzpatrick 1932; Epstein et al. 1997; Knapp et al. 1998), which comprises up to 80% of biomass in some native tallgrass prairies (Knapp et al. 1998), and is widely used in restorations. We focus on big bluestem because traits of the dominant species control grassland ecosystem functioning (Mokany et al. 2008). Thus, dominant grasses, such as big bluestem, are a key for recovery of the ecosystem during grassland restoration (Baer et al. 2002). Specifically, this study involves an investigation of natural variation in drought tolerance of ecotypes of big bluestem (*A. gerardii*) across the 1150 km longitudinal precipitation gradient of the Great Plains of the United States (latitude remains approximately constant). *Andropogon gerardii* has a wide natural distribution across the eastern United States (http://plants.usda.gov), with greatest dominance in the Great Plains (Epstein et al. 1997), thus lending support for likely natural variation and local adaptation across this climate gradient. Furthermore, this climate gradient and the grassland formation have been in place for the last 10 000 years (Axelrod 1985), allowing an excellent opportunity to test the extent of genetic adaptation to climate. On the other hand, this wide-ranging plant may to respond to heterogeneous environments (different precipitation regimes) through phenotypic plasticity, rather than genetic variation.

**Figure 1:**
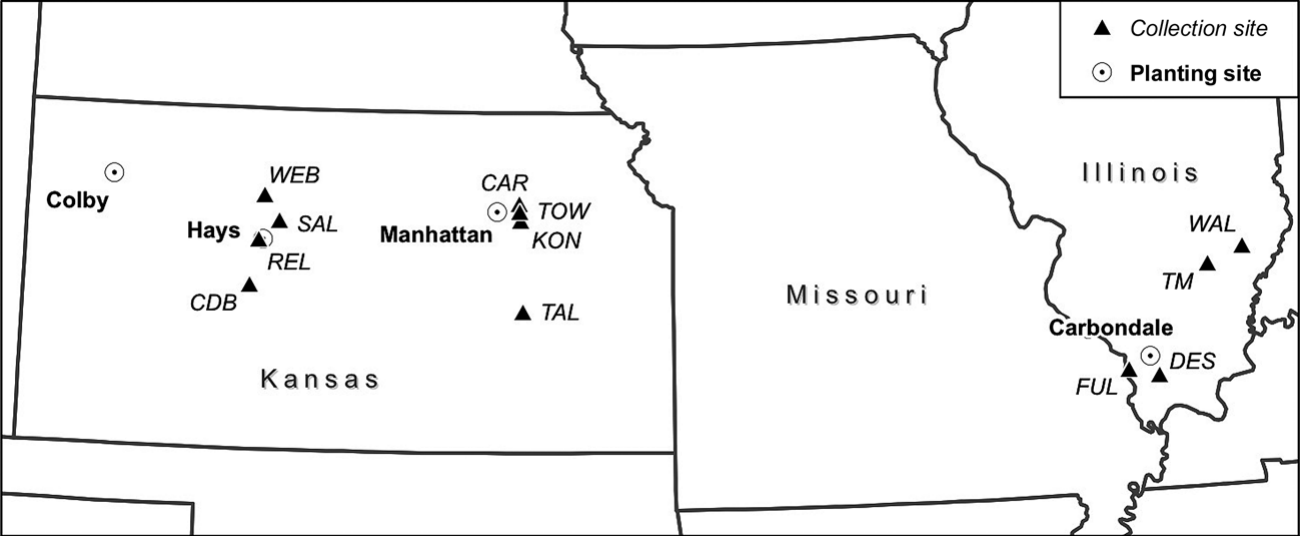
Location of reciprocal gardens planting and collections sites across the US Great Plains. White circle is planting site reciprocal garden location. Black triangles are the collection prairie for the seeds. For prairie population acronyms, see Table1. Colby, Kansas is the satellite reciprocal site to test the range of tolerance for big bluestem. Note that seeds were not collected in Colby.

Although *A. gerardii* has been extensively studied in ecological response to climate (Epstein et al. 1997; Knapp et al. 2001; Fay et al. 2002, 2003), community structure (Collins et al. 1998; Veen et al. 2008), and physiological performance (Silletti and Knapp 2001; Swemmer et al. 2006), studies are lacking on intraspecific variation across broad climate gradients. Within the Great Plains, *A. gerardii* occurs along a sharp mean annual precipitation (MAP) gradient in our study plots (Fig.1), from western KS (500 mm MAP) to Illinois (1200 mm MAP). We hypothesized *A. gerardii* ecotype responses will vary as a result of local adaptation to climate across a longitudinal precipitation gradient. Such knowledge of intraspecific variation in drought tolerance is critical to be able to predict and model grassland biome responses to climate change (Aspinwall et al. 2013). Our studies focusing on aboveground *A. gerardii* response complement others (Schultz et al. 2001; Johnson et al. 2010) showing *A*. *gerardii* ecotypes adapt belowground to their local soil arbuscular mycorrhizal fungal communities.

Adaptive variation and local adaptation among plant populations have been widely studied, both across large-scale climatic gradients (Clausen et al. 1940; McMillan 1959; Joshi et al. 2001; Bischoff et al. 2006; Ariza and Tielborger 2011; Savolainen et al. 2013) and over fine scales of environmental variation (Bradshaw 1984; Linhart and Grant 1996; Galloway and Fenster 2000; Montalvo and Ellstrand 2000; Volis et al. 2002; Etterson 2004; Knight et al. 2006; Lowry et al. 2009; Montesinos-Navarro et al. 2011). Recently, several adaptive variation studies have been performed within the context of restoration (Bischoff et al. 2006). In addition to local adaptation via genetic responses, plant species might perform well over a wide range of environments through phenotypic plasticity where a single, all-purpose genotype buffers the individual from the environment or changes the phenotypic expression of physiological and morphological traits to match environmental cues (Van Tienderen 1997). Plants with broad distribution, such as *A. gerardii*, may respond in part through phenotypic plasticity. Most frequently, some combination and interaction of phenotypic plasticity with genetically based adaptive variation are observed in plant species (Bradshaw 1984; Bazzaz 1986). Studies on adaptive variation take on urgency with restoration in changing climates, as it becomes imperative to predict how ecotypes within species may respond to climate change, either through plasticity, adaptive variation, or migration (Nicotra et al. 2010).

Our study has relevance for other grasslands worldwide, as grasslands are likely to experience increasing water stress (www/aqueduct.wri.org/atlas). Yet, the degree of adaptive variation within species across precipitation gradients is poorly characterized for most plants. Grassland covers one-third of continental North America (Bailey 1998), 40% of vegetated land worldwide, and is generally characterized by frequent droughts (Knapp et al. 1998, 2001; Knapp and Smith 2001; Craine et al. 2012). Drought limits plant productivity, especially in grasslands (Axelrod 1985; Knapp et al. 2001). US grasslands have recently experienced drought unprecedented since the 1930s (NOAA, 2014). Furthermore, one of the most important climatic changes predicted for grasslands over the next century is alteration in amount and timing of precipitation (Knapp et al. 2008; IPCC 2013). Consequently, understanding natural variation in drought tolerance of widespread grassland species, such as the foundation prairie grass *A. gerardii*, is particularly urgent, especially for restoration in the face of changing climates (Rice and Emery 2003; Harris et al. 2006). Results will inform land managers of suitable ecotypes for conservation plantings in the face of climate change (Rice and Emery 2003; Harris et al. 2006).

The overall objective of this study was to evaluate the extent to which vegetative performance of *A. gerardii* ecotypes varied across different climatic regions, thus potentially signaling local specialization, that is, genetic differentiation and adaptation of ecotypes to precipitation. Environmental variation at subcontinent scales results from different climatic conditions (Clausen et al. 1940; Schmid 1985; Weber and Schmid 1998; Bell et al. 2000; Etterson 2004). If environments with contrasting selection pressures (such as different climates) are compared in reciprocal transplant experiments, significant performance differences in the native versus transplanted gardens may be attributed to local adaptation via genetic differences among the ecotypes (Bradshaw 1984; Linhart and Grant 1996). Common garden experiments have been used to determine the relative contribution of genetics and environment to phenotypic variation (Kawecki and Ebert 2004) and are a powerful approach to investigate local adaptation among populations in plant communities (Clausen et al. 1940; Etterson 2004; Knight et al. 2006; Oyarzabal et al. 2008; Lowry et al. 2009).

Here, we used a reciprocal common garden approach containing four sites that span 1150 km of tallgrass prairie. We hypothesized precipitation differences across this environmental gradient would exert selection pressures strong enough to overcome gene flow in this wind-pollinated and wind-dispersed species, resulting in local adaptation to ‘home’ environmental conditions (Fig.1). If ecotypes are genetically fixed and locally adapted to climate, restoring with resident plants would be recommended (McKay et al. 2005) and remains the most conservative strategy for restoration. If drought adapted ecotypes are identified, restoring with mixed ecotypes to anticipate and mitigate future climate change might be considered (Broadhurst et al. 2008). If plants are phenotypically plastic in response to climate, then restoring with the resident plants would not be necessary and, in fact, would be discouraged, especially if resident plants were derived from small, fragmented prairie populations that may suffer from genetic erosion (Bijlsma and Loeschcke 2012).

Our experiment is novel in that we assembled big bluestem ecotypes and multispecies communities, simulating a more realistic test of ecological adaptation, as well as restorations that might occur in practice (Baer et al. 2014). Our design is nearly unprecedented in scope in that the experiment incorporates biotic (competitors) and abiotic (climate) factors in driving adaptation. Biotic factors are rarely tested (Bischoff et al. 2006; Ariza and Tielborger 2011); in fact, local adaptation studies are often conducted under reduced competition (monocultures, spaced plantings, weeding), thus neglecting the local plant community as a potential biotic driving force of local adaptation (Bischoff et al. 2006; Ariza and Tielborger 2011). Furthermore, we planted these restored communities from seed rather than planting seedlings, similar to a grassland restoration. Seedlings grown under standardized greenhouse conditions and then planted in the field (Bischoff et al. 2006) might ignore the potential for adaptive population differentiation in seed survival, dormancy, and germination phases (Nagy and Rice 1997; Keller and Kollmann 1999; Galloway and Fenster 2000; Bischoff et al. 2006; Donohue et al. 2010).

We also sought to make predictions about the extent to which *A. gerardii* could extend its range into drier climates to make predictions about how this species might respond to harsher environmental conditions as predicted by climate change models. Common gardens are becoming widely used in a ‘chronosequence context’ (Etterson 2004; Shaw and Etterson 2012) in which a climate gradient is used as a proxy for the projected climate and to make predictions about the extent of range expansion. To test this, in addition to the reciprocal gardens, we planted ecotypes of *A. gerardii* into the westernmost and driest region of its distribution in the Great Plains (Colby, KS) to test the extent to which ecotypes might respond to increasingly dry conditions (Weltzin et al. 2003), as predicted by climate models (IPCC 2013) and as a surrogate for ecotypic response under future extreme dry conditions (Shaw and Etterson 2012).

This study addressed the following questions: (i) Are *A. gerardii* ecotypes locally adapted to environment across the precipitation gradient? (ii) What is the relative role of genotype and environment in controlling these differences? (iii) How will different ecotypes of *A. gerardii* respond under different climatic conditions, especially precipitation, in native and reciprocally transplanted environments? (iv) How can we use this information to make predictions regarding restoration of *A. gerardii in* current and changing climates? To answer these questions, we measured responses of *A. gerardii* over several timescales, ranging from instantaneous gas exchange to medium-term responses (chlorophyll absorbance) to establishment and cover over 2 years. This combination of short-, medium-, and long-duration responses should provide a comprehensive understanding of *A. gerardii* ecotype responses to climate across the Great Plains.

Specifically, we hypothesized that (i) regional ecotypes (originating from central and eastern KS and southern Illinois, i.e. CKS, EKS, and SIL, respectively) would perform the best relative to nonresidents in their home environment as measured by gas exchange (higher stomatal conductance, *g*_s_, and higher photosynthesis, *A*), higher chlorophyll absorbance, greater seedling establishment, and increased vegetative cover, (ii) the central KS (CKS) ecotype, collected from xeric source populations in the west, would maintain higher *g*_s_, *A*, and chlorophyll absorbance, and have greater plant cover compared to ecotypes from more mesic source populations in the east (EKS, SIL) when all were grown in xeric planting sites, (iii) mesic sites (Manhattan, KS and Carbondale, IL) were expected to have greater plant cover, irrespective of ecotype, and plants growing there should have higher *A* and *g*_s_, and (iv) if local adaptation is strongly enforced by climatic selection for drought ecotypes in arid areas, then the CKS ecotype should outperform other ecotypes when planted in Colby, KS, the most arid end of the gradient. Here, we present results from the first 2 years of vegetative performance as an indication of fitness potential in the ongoing reciprocal garden restoration experiment.

## Materials and methods

### Plant materials and seed collection sites

Seed of *A. gerardii* was collected by hand during autumn 2008, from three climatically distinct regions along a precipitation gradient from CKS, EKS, and southern IL (Table1, Fig.1), roughly corresponding to the ecoregions (Bailey 1998) of Great Plains Steppe (CKS) and Great Plains Steppe/Prairie Parkland Temperate (EKS) and Prairie Parkland Temperate (southern IL). In each region, seeds were collected from four populations, which jointly defined a regional ecotype (referred to as CKS for central KS, EKS for eastern KS, and SIL for southern IL). We have previously characterized the ecological fidelity of the source plant populations to their respective regional ecotype (M. Galliart, unpublished data). That is, morphological traits of the founding populations vary among ecotypes, but populations do not differ within ecotypes, thus confirming the regional nature of the ecotypes. Populations originated from intact, never restored prairies within a 80 km radius of the reciprocal garden planting site (Table1). Seeds from each population were collected on at least three dates during autumn 2008. All big bluestem seed was analyzed for seed filling, germination, and dormancy to determine percent live seed by the KS Seed Crop Improvement Center (Manhattan, KS, USA). After accounting for percent live seed, seeds from four populations within each ecotype were mixed in equal quantities for the final seed mix used in plot establishment. Plots were planted in spring 2009.

**Table 1:** Location of seed collection populations from the three ecoregions

Ecotype	Collection site	County	Latitude (N)	Longitude (W)	Elevation (m)	Size in hectares
Western Kansas	Relict Prairie	Ellis	38°51′	99°22′	659	14
	Webster Res.	Rooks	39°24′	99°32′	606	356
	Saline Expt. Range	Ellis	39°02′	99°14′	641	970
	Cedar Bluffs Res.	Trego	38°45′	99°46′	688	445
Central Kansas	Carnahan Cove St. Pk.	Pottawatomie	39°20′	96°38′	389	100
	Konza Prairie	Riley/Geary	39°05′	96°36′	366	1411
	Tallgrass Prairie Nat. Pk.	Chase	38°25′	96°33′	392	4408
	Top of the World Pk.	Riley	39°13′	96°37′	379	61
Southern Illinois	Desoto Railroad Prairie	Jackson	37°51′	89°14′	119	0.4
	Twelve Mile Railroad Prairie	Effingham, Fayette, and Marion	38°46′	88°50′	160	28
	Fults Hill Prairie	Monroe	37°58′	89°48′	215	213
	Walters Prairie	Jasper	38°59′	88°09′	150	5

### Reciprocal garden design and plot establishment

Each ecotype was reciprocally seeded into plots at four sites, representing mean precipitation ranging from 500 to ∼1200 mm year^−1^: Colby, KS (500 mm); Hays, KS (580 mm); Manhattan, KS (871 mm); and Carbondale, IL (1167 mm) (Table2, Fig.1). Although we did not have a Colby, KS ecotype, the Colby site was added to test the tolerance of all three ecotypes to extremely arid environments (Table2), similar to what *A. gerardii* might experience with future warming and drying.

**Table 2:** Selected long-term and seasonal environmental variables used to describe the planting sites

Reciprocal garden planting sites	Ave temp, °C (max, min)	Elevation (m)	MAP, since 1961 (cm)	2010 2009 ppt (cm)	Growing season ppt (April 1 –August 31, 2010)	Ppt of driest year (cm, year)	GDD ave	GDD 2010	PET (cm)	Aridity index (PET-ppt)
Colby, KS	10.5 (18.4, 2.6)	972	50.47 (±11.77)	47.72	39.21	28.37 (1967)	3167	3461	144	97
KSU Ag. Res. Center				67.81						
Thomas County										
39°23′N,										
101°04′W										
Hays, KS	12 (19.3, 4.7)	603	58.22 (±13.13)	58.29	48.84	36.27 (1988)	3799	4193	139	81
KSU Ag. Res. Center				55.12						
Ellis County										
38°51′N,										
99°19′W										
Manhattan, KS	13 (19.6, 6.4)	315	87.15 (±20.04)	84.68	67.23	39.16 (1966)	4156	4105	127	41
USDA Plant Material Center				98.27						
Riley County										
39°08′N,										
96°38′W										
Carbondale, Illinois	13.3 (19.5, 7.1)	127	116.73 (±24.76)	80.31	47.01	67.38 (1963)	4087	4474	99	−18
SIU Agronomy Center				154.58						
Jackson County										
37°73′N,										
89°22′W										

Ppt, precipitation; PET, potential evapotranspiration; MAP, mean annual precipitation.

Growing degree days was calculated as GDD = [(AT_max_ + *T*_min_)/2]−50; if GDD is <0, set to 0.

In terms of pre-experiment conditions and vegetation, the four garden sites were all under agricultural cultivation prior to the reciprocal garden establishment, similar to conditions expected on restorations. The three sites in Kansas (Colby, Hays, and Manhattan) were cultivated for >30, 70, and 74 years prior to seeding, respectively. The easternmost site, Carbondale, IL, had a more variable land-use history and was cultivated since 2004. In Colby, wheat and sorghum were the most recent crops, whereas sudangrass (*Sorghum bicolor* (L.) Moench ssp. *drummondii* (Nees ex Steud.) de Wet & Harlan) and sorghum (*S. bicolor* (L.) Moench ssp. *bicolor*) were most recent in Hays. Soybeans (*Glycine max* (L.) Merr.) were planted in Manhattan for 5 years prior to the study. The Carbondale site was cultivated to corn (*Zea mays* L.) the year prior to seeding. Soils were all classified as loams; soil at the eastern three sites was classified as silt loam, and Colby as silt clay loam. Other chemical properties varied somewhat from site to site (Goad 2012). The Manhattan site contained very sandy, nutrient-poor soil [as indexed by low percent carbon (C), percent nitrogen (N), and cation exchange capacity] (Goad 2012).

The experiment was a reciprocally transplanted, randomized complete block design with four blocks per site. Within a site, each block consisted of three plots with each plot seeded to a regional ecotype. Plots were plowed within a week prior to garden establishment (no herbicide applied) and sown to each regional ecotype in June 2009, using a 70:30 ratio of live C_4_-grass to C_3_-grass and forb seed (Table3). Seeds were mixed with damp sand to aid in homogenous dispersal. Sand and seed were hand-broadcast into the experiment sites and raked into the soil. Total seed density for each plot was 580 seeds m^−2^, as recommended for prairie restoration (Packard and Mutel 1997). *Andropogon gerardii* was planted at a density of 270 live seeds m^−2^. Seeds of eight other species (Table3) were added to maintain characteristic functional group structure and competitive relationships of tallgrass prairie. Planted seeds of all species, except Indiangrass (*Sorghastrum nutans*) for which resident seeds were collected and planted at each site, were purchased from a commercial supplier (Ion Exchange Inc., Harpers Ferry, IA, USA). Seeds sold by this supplier were sourced from across the Great Plains and grown for production in the Harpers Ferry nurseries. Species composition of the planted seed as well as seeding rate is typical for prairie restorations. While we would have preferred collecting local seed of all species used in the experiment, this was intractable due to the plot size and overall size of the experiment (each reciprocal garden site was approximately the size of a football field). Furthermore, broad-scale restorations typically use commercial seed making our experiment relevant for restorations. Additionally, plants of volunteer species from regional propagule sources also established on their own in the garden sites, making the composition of the community at each garden site a mix of volunteer and planted species (Goad 2012; Wilson 2013). Each plot was separated by a 4–6 m buffer strip.

**Table 3:** Seeding rates and species used for reciprocal garden plots. Nomenclature follows USDA plants database (USDA, NRCS, 2015)

Planted species	Family	Functional group	Seeding rate (live seed m^−2^)
*Andropogon gerardii* Vitman	Poaceae	C_4_ grass	270
*Sorghastrum nutans* (L.) Nash	Poaceae	C_4_ grass	70
*Elymus canadensis* L.	Poaceae	C_3_ grass	30
*Asclepias tuberosa* L.	Asclepiadaceae	Forb	30
*Chamaecrista fasciculata* (Michx.)			
Greene	Fabaceae	Legume	30
*Dalea purpurea* Vent.	Fabaceae	Legume	30
*Monarda fistulosa* L.	Lamiaceae	Forb	30
*Oligoneuron rigidum* (L.) Small	Asteraceae	Forb	30
*Penstemon digitalis* Nutt. ex Sims	Scrophulariaceae	Forb	30
*Ruellia humilis* Nutt.	Acanthaceae	Forb	30
Total seeds m^−2^			580

### Environmental variables

**Figure 2:**
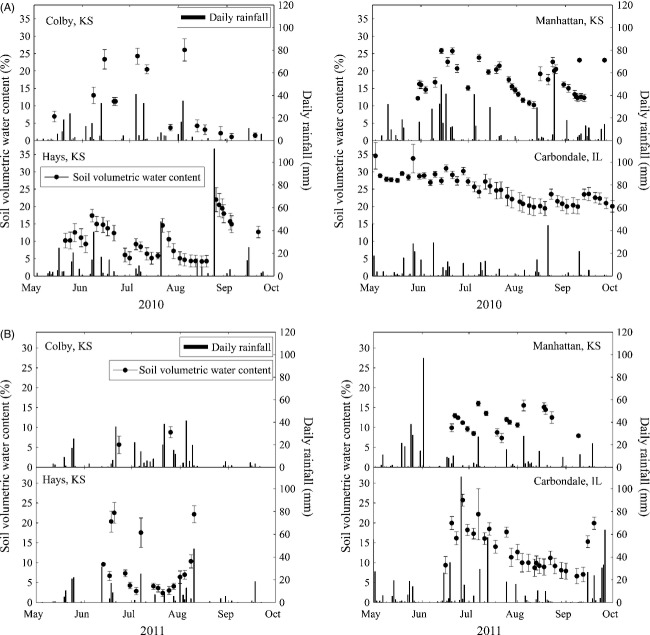
Daily rainfall and soil moisture at the reciprocal garden planting sites for (A) 2010 and (B) 2011. Daily rainfall events indicated by bars (right *Y* axis), volumetric water content indicated by line graphs (left *Y* axis). Points are means of four probes per site ± SE.

We monitored soil moisture at each site and compiled precipitation data for each region. Soil moisture from 0 to 20 cm depth was monitored using four EC-20 soil moisture probes (Decagon Devices, Inc., Pullman, WA, USA) that were arranged diagonally across the plots at each site, with one probe per block. Probes were positioned at the beginning of the growing season (early May) and measured every 3–4 days in all sites with the exception of Colby, KS (Fig.2). Data on daily precipitation from each site were collected at either local agricultural research stations (KSU in Hays, Colby), USDA Plant Material Center in Manhattan, and at the Southern Illinois University Agricultural Research Facility or nearby NOAA weather stations. In summer of 2009, shortly following seeding, natural precipitation was supplemented with 25 mm at the Hays site to alleviate a severe deficit during plot establishment. Supplemental water was not added thereafter. This supplement increased precipitation to the average received for that time of year, based on historical records. For long-term average precipitation and temperature data, we used the NOAA database dating to 1961. All of the reciprocal garden sites are situated at approximately the same latitude and within USDA hardiness zone 5 (−28.8 to −23.4°C average annual minimum temperature).

At each planting site, we calculated growing degree days (GDD) to account for phenology differences across the 1150 km gradient. GDD was later used as a covariate in statistical analyses. In this manner, we could explore differences among sites for a given GDD, thus accounting for percent of the elapsed growing season. We developed GDD accumulations for big bluestem, with modifications from maize (Russelle et al. 1984; Neild and Newman 1990). GDD, an index of days appropriate for *A. gerardii* growth based on temperature, were calculated for each site and each day of the growing season with the following formula:

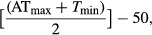
 where AT_max_ is the maximum adjusted temperature, with temperatures greater than 35°C set equal to 35°C, and *T*_min_ is the temperature minimum for each day. Negative results were adjusted to 0. We summed GDD daily at each site for all years from 1961 through 2011 to determine average annual GDD and averaged these data over 50 years to determine long-term averages (Table2). We also calculated GDD for the year 2010, the main measurement year for this study. Potential evapotranspiration (PET) was calculated from free water surface evaporative demand and MAP (J. K. Koelliker, personal communication). Aridity index was calculated as PET precipitation. We used the USDA soils survey classifications and all soils in the gardens were silt loams. Chemical analyses were performed on the plot soils (one core from each block) (Table4; Goad 2012).

**Table 4:** Soil characteristics from soils collected at the planting sites (*n* = 4)

Site	Colby, KS	Hays, KS	Manhattan, KS	Carbondale, IL
Silt loam type	Ulysses	McCook	Belvue	Stoy
CEC	24.6	25.1	8.5	13.7
% sand	8.5	21.5	41.0	7.5
% silt	60.0	58.5	51.0	78.5
% clay	31.5	20.0	8.0	14.0

### Response variables measured on all ecotypes in all sites

#### Establishment and vegetative cover

Establishment and cover are intended to represent long-term measures of plant performance. Establishment of *A. gerardii* ecotypes was measured in October 2009 at all sites. Quadrats (0.25 m × 0.25 m) were randomly positioned in four locations within each plot to count *A. gerardii* seedlings. The purpose of these measurements was to quantify establishment in the first growing season following seeding in June 2009. No other species were counted. Establishment was evaluated using a one-time measurement at each site at the end of the growing season in mid-October. The majority of the measurements were carried out the following year to minimize maternal effects.

For each site, vegetation cover was measured twice during the 2010 growing season, in early June and late August. To estimate percent cover, a 1.0 m^2^ quadrat was used, the quadrant consisted of one intersection every 10 cm for a total of 81 intersections (Greig Smith 1983). At every intersection, occurrence of *A. gerardii*, other grass, forb, or bare ground was recorded. In each plot, four nonoverlapping quadrats were randomly placed at least 50 cm from the edge to minimize edge effect. In addition to plant cover, biomass (ANPP) of each species in the plots (*A. gerardii*, eight planted species, as well as volunteers arriving from the regional propagule pool) was harvested at the end of each growing season, weighed, and analyzed separately to provide an estimate of community-level responses. These data are reported separately (Goad 2012; Wilson 2013). This study focuses mainly on the big bluestem ecotype response.

#### Chlorophyll absorbance using SPAD meter

To characterize the regional ecotypes in terms of plant performance, we measured chlorophyll absorbance with a SPAD 502 (Konica Minolta, Osaka, Japan) 3–4 times during summer 2011. SPAD represents a medium-term (weeks) response variable. Chlorophyll absorbance (Lin et al. 2010) was intended to complement the instantaneous gas exchange measurements and long-term cover measurements to understand the photosynthetic biology of big bluestem throughout the growing season. We randomly selected six plants from each plot at each time. On each plant, we measured chlorophyll absorbance on four leaves chosen randomly from the four compass directions.

#### Gas exchange

Gas exchange measures are intended to represent instantaneous measures of plant performance. We measured photosynthesis (*A*, μmol CO_2_ m^−2^ s^−1^), stomatal conductance (*g*_s_, mol H_2_O m^−2^ s^−1^), transpiration (*E*, mmol H_2_O m^−2^ s^−1^), internal CO_2_ (*C*_i_, ppm), and intrinsic water-use efficiency (WUE = *A*/*g*_s_) from six plants randomly selected from each plot using an LI-6400 (Li-Cor Inc., Lincoln, NE, USA). Gas exchange was measured on a young, fully expanded leaf with CO_2_ levels at 385 ppm, humidity and temperature at ambient levels, and photosynthetically active radiation (PAR) at 1500 μmol photons m^−2^ s^−1^. Measurements were made on sunny days between 10:00 and 15:00 h to minimize adjustment time to leaf chamber conditions. Measurements were made when photosynthesis and stomatal conductance had stabilized (<3% coefficient of variation). Gas exchange was measured in the plants three times during the 2010 growing season: early in the growing season, mid-season, and late in the growing season. Different instruments were used to measure gas exchange at each site. We tested each instrument against a gas standard, and values were accurate within 2.8% of actual values.

### Statistical analysis

To assess establishment, a generalized linear mixed model was fit to the seedling count response measured in October 2009 using a negative binomial distribution with a log link function. The model included the fixed effects of planting site (Colby, Hays, Manhattan, KS, and Carbondale IL), ecotype (CKS, EKS, and SIL), and their two-way interaction. Random effects of block were nested within site and crossed with ecotype to recognize the appropriate experimental unit for each fixed effect factor. Parameter estimation was conducted using residual pseudo-likelihood with Newton Raphson with ridging as the optimization technique. Kenward-Roger’s procedure was used to estimate degrees of freedom and to make the corresponding adjustments in estimation of standard errors.

Vegetative cover, SPAD, and gas exchange were measured multiple times through the season at the four sites. In all cases, a generalized linear mixed model was fit to the response variable. The statistical model included the fixed effects of planting site (Colby, Hays, Manhattan, KS and Carbondale IL), ecotype (CKS, EKS, SIL), and their two-way interaction. Proportion GDD in linear and quadratic polynomial form (except for cover, for which only the linear form was included in the model) was fit to the model as a covariate, along with all two- and three-way interactions with the fixed effects of site and ecotype. Due to significant high-order interactions involving GDD for many of the response variables, comparisons between sites and ecotypes were conducted at selected values of GDD that lay within the range of the groups intended for comparison. More specifically, for cover, comparisons were conducted at 0.35 and 0.65 GDD of the growing season elapsed, as this was the range of GDD for which cover was observed in all sites. Chlorophyll absorbance was compared between sites at 0.50, 0.60, and 0.70 GDD of the growing season elapsed, as (0.50, 0.70) was the range of GDD for which chlorophyll absorbance was observed in all sites. In turn, for gas exchange measures, comparisons were conducted between 0.40 and 0.70 GDD, as this was the range of GDD across sites for data collection on gas exchange.

Random effects included block nested within site and also crossed with ecotype, to recognize their appropriate experimental units. For the models fit to *C*_i_ and WUE, the random effect of block nested within site yielded a variance estimate that converged to zero and was thus removed from the model. Furthermore, for *C*_i_ and WUE, the Bayesian information criterion indicated improved model fit when heterogeneous residual variances were specified for each site. Variance components were estimated using residual maximum likelihood. Kenward-Roger’s procedure was used to estimate degrees of freedom and make the corresponding adjustments in estimated standard errors. Model assumptions were evaluated using externally studentized residuals and were considered to be appropriately met.

Statistical analyses described thus far were implemented using the GLIMMIX procedure of sas (Version 9.2; SAS Institute, Cary, NC, USA). Least square mean estimates, as well as estimated standard errors or 95% confidence intervals, are reported for each response analyzed. Comparisons of interest were adjusted using Tukey–Kramer (marginal effect differences) or Bonferroni (simple effect differences) to avoid inflation of Type I error rate due to multiple comparisons.

For each ecotype, linear regression models were fitted to the response percent cover for big bluestem (average of measurements early and late in the season for a given block) as a function of historic mean annual precipitation (MAP1961). For each ecotype, the statistical model evaluated linear, quadratic, and cubic effects of MAP1961 on percent cover. Variance components were estimated using the anova method to compute a coefficient of determination *R*^2^ for each ecotype. Model assumptions were evaluated using studentized residuals and were considered to be reasonably met. The statistical models for each ecotype were fitted using the GLM procedure of sas (Version 9.2; SAS Institute). Parameter estimates and corresponding standard errors are reported.

## Results

### Establishment and survival: seedling counts

**Figure 3:**
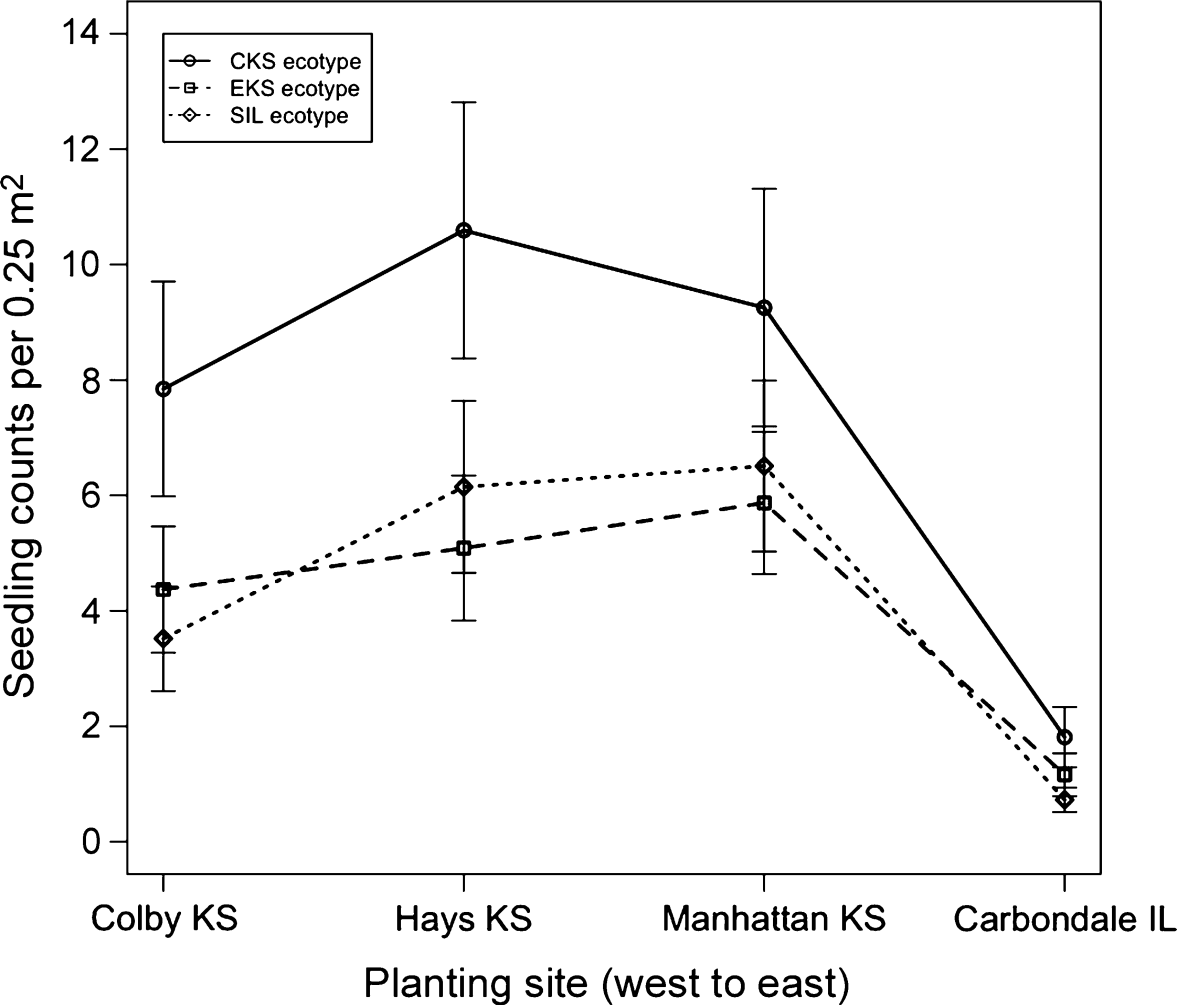
Seedling counts (least squares mean estimates ± SE) at the end of the first growing season for big bluestem ecotypes in planting sites across the Great Plains precipitation gradient. Counts made in 0.25 m^2^ quadrats, *n *=* *4 per plot. CKS = central KS ecotype (circle), EKS = eastern KS ecotype (square), SIL = southern Illinois ecotype (diamond).

In October 2009, at the end of the growing season at all planting sites, there was no evidence for any two-way interaction between ecotype and planting site on seedling counts (*P* = 0.832). However, a main effect of ecotypes was identified on seedling counts (*P* = 0.002; Fig.3, Table S1). At all planting sites, the CKS ecotype had more seedlings than either the EKS or SIL ecotypes, which were not significantly different from each other. Similarly, seedling counts differed between planting sites for all ecotypes (*P* < 0.001; Fig.3, Table S1), where seedling counts were fewest in Carbondale relative to the remaining planting sites, which were not significantly different from each other. In summary, the CKS ecotype showed greatest establishment relative to the other ecotypes, regardless of planting site. This relative advantage of the CKS ecotype, which seemed to be comparable across sites (i.e. no evidence for any ecotype-by-site interaction), was of particular interest at the drier planting sites in Hays and Colby.

### Vegetative cover

**Figure 4:**
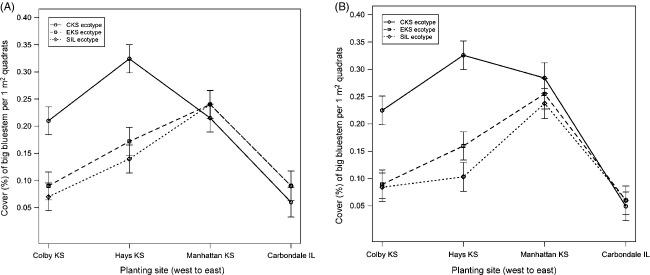
Vegetative cover (least square mean estimates ± SE) for each ecotype at planting sites across the Great Plains precipitation gradient at 0.35 GDD (4A) and 0.65 GDD (4B), corresponding to the earliest and latest time point in the growing season (i.e. 35%, 65%) for which inference is granted in all four sites. CKS = central KS ecotype (circle), EKS = eastern KS ecotype (square), SIL = southern Illinois ecotype (diamond).

By 2010, there was evidence for a two-way interaction between site and ecotype on big bluestem cover (*P* = 0.001), with the CKS ecotype having disproportionately greater cover at the drier Hays and Colby planting sites, relative to the other ecotypes (Fig.4, Table S1). This relative advantage of the CKS ecotype at drier sites was apparent throughout the growing season, both at 0.35 GDD (*P* < 0.003) and at 0.65 GDD (*P* < 0.001). These values of GDD were selected for inference as they were the most extreme within the observed range of GDD for all planting sites. In Hays and Colby, the CKS ecotype had significantly greater cover than the SIL or EKS ecotypes, regardless of GDD (Fig.4). In the Carbondale and Manhattan planting sites, there was no evidence for differences in vegetative cover between ecotypes, regardless of GDD (*P* > 0.60). SIL and EKS ecotypes had cover that was not significantly different from each other at any of the sites throughout the growing season (*P* > 0.30). In general, total plant cover (all species combined) at all sites was high >80%, suggesting good establishment of focal grass species *A. gerardii*, as well as plants from the eight seeded species and plants of volunteer species arriving from the regional propagule pool (Goad 2012). This suggests that reduced competition in the xeric sites is not the mechanism for increased cover of *A. gerardii* in dry sites in Colby and Hays.

**Figure 5:**
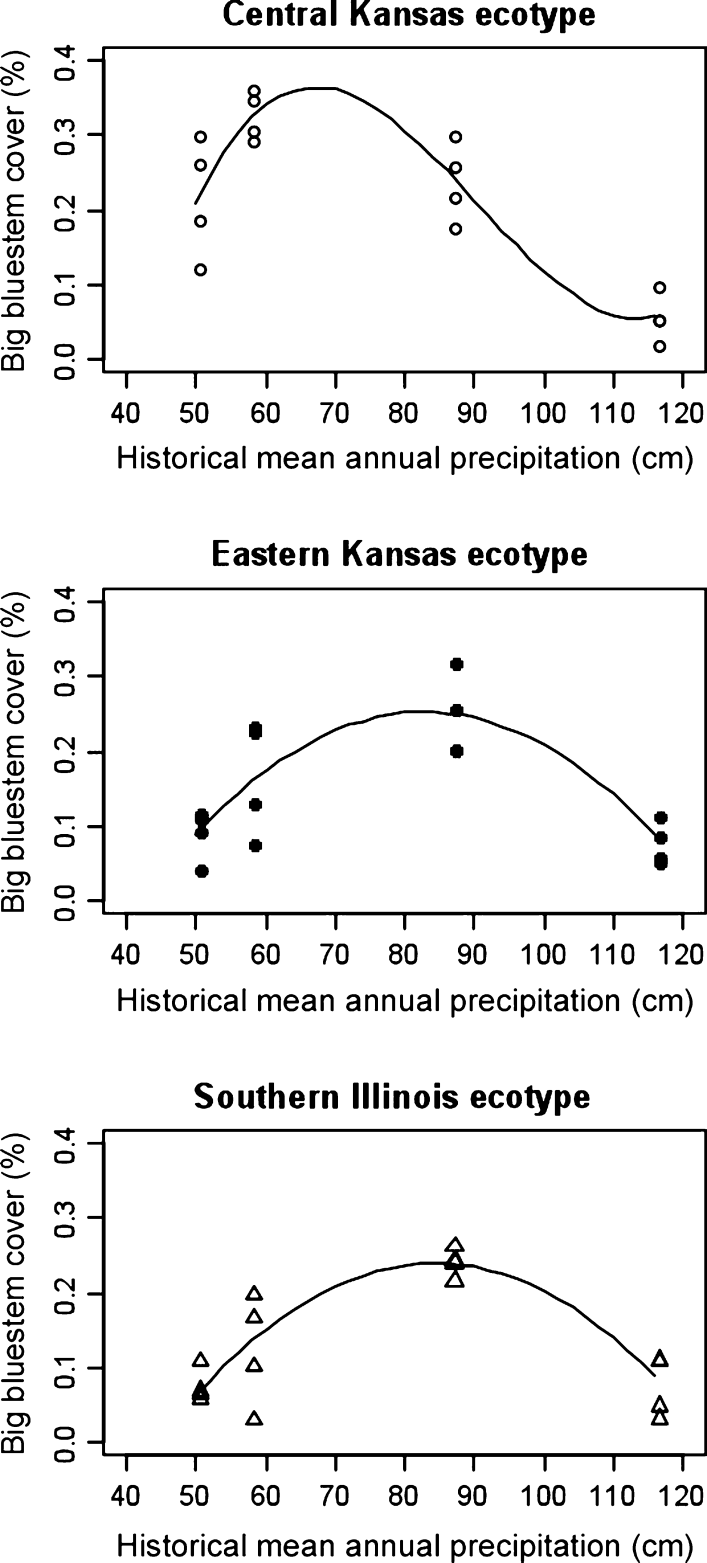
Vegetative cover of big bluestem ecotypes (combined 2010 data) as a function of long-term historical mean annual precipitation (cm) for the corresponding planting sites (50 cm: Colby, KS; 58 cm: Hays, KS; 88 cm: Manhattan, KS; 115 cm: Carbondale, IL). Points indicate actual observations in four plots per planting site, line indicates fit.

To further investigate the differential performance of the ecotypes across the climatic gradient, we plotted vegetative cover of each ecotype as a function of the historical mean annual rainfall at each site (Fig.5). The nature of the association between cover and precipitation was fundamentally different across ecotypes. For the CKS ecotype, the relationship between percent cover and historic MAP was best described by a cubic polynomial function (*P* < 0.035), with greatest cover observed in the drier planting locations. For EKS and SIL ecotypes, the functional relationship between percent cover and historic MAP was characterized as quadratic in shape (*P* < 0.0001 in each case). This implied that for the more mesic EKS and SIL ecotypes, greatest cover occurred at planting sites with intermediate precipitation levels. The coefficient of determination *R*^2^ obtained from the fitted linear regressions indicated historic MAP accounted for 82.1%, 69.1%, and 74.1% of the variability in percent cover of big bluestem for CKS, EKS, and SIL ecotypes, respectively.

### SPAD chlorophyll absorbance

**Figure 6:**
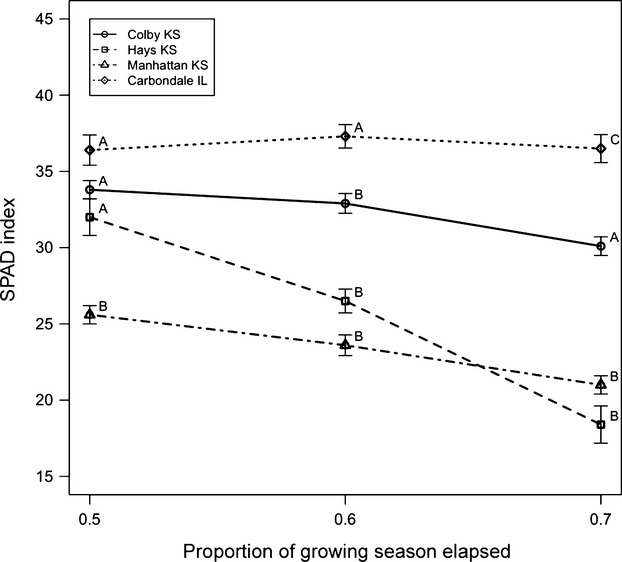
SPAD values (least squares mean estimates ± SE) for each planting site as a function of proportion of growing season elapsed. Higher numbers reflect higher chlorophyll content. A, B letters indicate significant differences among sites at a given GDD. Diamond = Carbondale, circle = Colby, square = Hays, triangle = Manhattan.

There was no evidence for any three-way interaction between planting site, ecotype, and GDD. However, a two-way interaction was apparent between planting site and the quadratic term for proportion GDD on the SPAD chlorophyll absorbance (*P* < 0.001, Fig.6, Table S2). This is indicative of a site-specific quadratic change in SPAD values over the growing season that was applicable to all ecotypes. Pairwise comparisons between sites (Fig.6) indicate that later in the season (i.e. GDD ≥ 0.6), SPAD index was maximum in Carbondale and minimum at the Manhattan and Hays sites, the latter of which were not significantly different from each other. For GDD ≥ 0.6, the Colby site showed SPAD values intermediate, yet significantly different from the remaining sites. Earlier in the growing season (i.e. GDD < 0.6), the Manhattan planting site showed lower SPAD values relative to the other planting sites, which were not significantly different from each other. Overall, each site showed a quadratic decline in SPAD values as the growing season progressed, except for Carbondale, where SPAD remained high throughout the season (Fig.6).

**Figure 7:**
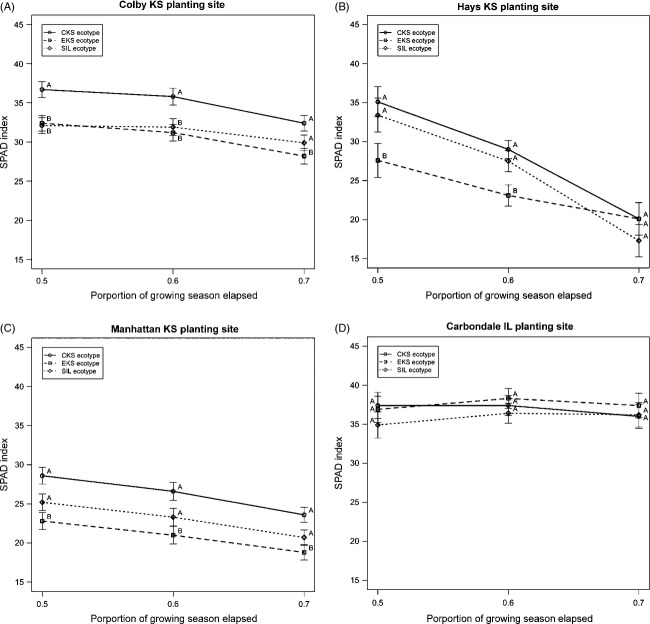
SPAD index values (least squares mean estimates ± SE) for selected ecotypes plotted over proportion of the growing season elapsed at each planting site (Colby, Hays, and Manhattan, KS and Carbondale, Illinois). Higher numbers reflect higher chlorophyll content. CKS = central KS ecotype (circle), EKS = eastern KS ecotype (square), SIL = southern Illinois ecotype (diamond). A, B letters indicate significant differences between ecotypes within a planting site and at a given stage of the growing season.

Pairwise comparisons between ecotypes at each site indicated that, for most of the growing season, the CKS ecotype generally had greater SPAD values (Fig.7, *P* ≤ 0.04) than other ecotypes at the Colby planting site. At the Hays planting site, the CKS and SIL ecotypes were not significantly different from each other, yet had consistently greater SPAD values than the EKS ecotype (*P* ≤ 0.04) in the earlier part of the growing season. Similarly, at the Manhattan planting sites, the CKS and SIL ecotypes were not significantly different from each other and had consistently greater SPAD values compared to the EKS ecotype (*P* < 0.002). No evidence for ecotypes differences in SPAD values was apparent at the Carbondale planting site at any point during the growing season (*P* ≥ 0.89).

### Gas exchange

#### Photosynthesis (*A*)

**Figure 8:**
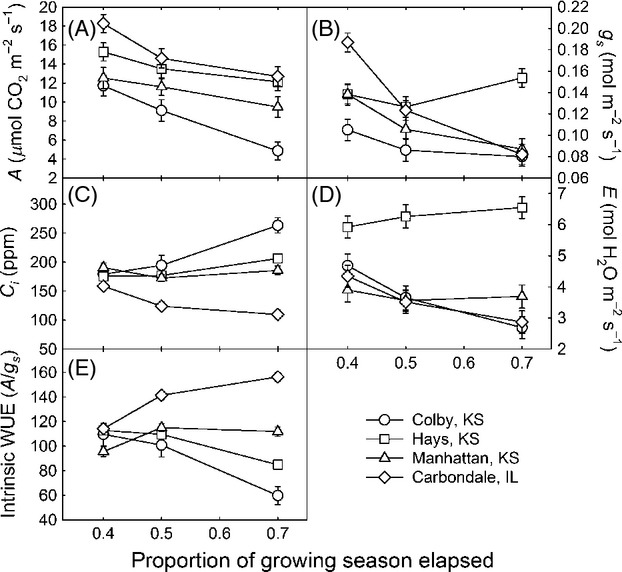
*A* Photosynthesis (A), *g*_s_ Stomatal Conductance (B), Internal CO_2_ (*C*_i_) (C), *E* Transpiration (D), and Intrinsic water-use efficiency (WUE, E, calculated as *A*/*g*_s_) of *Andropogon gerardii* ecotypes grown in Colby, KS (circle), Hays, KS (square), Manhattan, KS (triangle), and Carbondale, IL (diamond) planting sites in 2010 (least squares mean estimates ± SE) plotted over growing degree days, expressed as a proportion of elapsed season. All measures were made at 1500 μmol photons m^−2^ s^−1^. Points are means of four replicate plots ± SE.

Leaf-level photosynthesis (*A*, μmol CO_2_ m^−2^ s^−1^) was mostly influenced by site and stage of the growing season, as a two-way interaction was apparent between planting site and GDD in quadratic form (*P* = 0.006, Fig.8A, Table S2). For all ecotypes, differences in photosynthesis between sites varied dynamically through the growing season, with greatest *A* early in the growing season and decreasing *A* as the season progressed; the seasonal decline was particularly marked in the driest planting site at Colby. Early in the growing season, *A* was greater at the wettest planting site in Carbondale relative to Manhattan and Colby (*P* ≤ 0.013). By 0.7 GDD, *A* was significantly lower at the driest site in Colby, KS relative to the other sites (*P* ≤ 0.045).

Pairwise differences between ecotypes were identified at drier planting sites, where the CKS ecotype differed in photosynthetic rates from the more mesic ecotypes from EKS and SIL. More specifically, at 0.3 GDD, photosynthesis was higher in the CKS ecotype (20.7 ± 1.5) compared to the SIL ecotype (15.1 ± 1.5) at the Hays site (*P* = 0.032). At 0.6 GDD in Colby, photosynthesis was marginally greater for the CKS ecotype (9.9 ± 1.5) compared to the EKS ecotype (4.7 ± 1.5, *P* ≤ 0.05).

#### Stomatal conductance (*g*_s_)

Stomatal conductance to water vapor (*g*_s_, mol H_2_O m^−2^ s^−1^) showed patterns similar to those observed in *A* and differed mostly by planting site and growing season (Fig.8B, Table S2), where site-specific quadratic effects of GDD on *g*_s_ were apparent (*P* = 0.001). Regardless of ecotype, differences in stomatal conductance between sites varied dynamically through the growing season. During the early growing season, *g*_s_ was greater at the wettest site in Carbondale compared to all other sites (*P* ≤ 0.003). As the growing season progressed, *g*_s_ decreased quadratically at Carbondale, Colby, and Manhattan planting sites. In the Hays planting site, *g*_s_ showed a slight decrease followed by an increase so that by late in the growing season, *g*_s_ in Hays was significantly greater than in all other sites (*P* < 0.001). Pairwise differences between ecotypes were identified only at the Hays site for 0.3 GDD, with the CKS ecotype showing greater *g*_s_ stomatal conductance (0.2 ± 0.01 mol H_2_O m^−2^ s^−1^) compared to the more mesic ecotypes from EKS (0.15 ± 0.01) and SIL (0.15 ± 0.01) in these conditions (*P* ≤ 0.026).

#### Intercellular CO_2_ (*C*_i_)

Intercellular CO_2_ concentrations (*C*_i_, ppm) showed a site-specific quadratic pattern over the growing season (Fig.8C), regardless of ecotype (*P* = 0.023). Early in the growing season (GDD = 0.4), *C*_i_ in Manhattan was greater than in Carbondale (*P* = 0.019), whereas the remaining sites were intermediate and not significantly different from either. As the growing season progressed, *C*_i_ in Colby and Hays increased whereas *C*_i_ in Carbondale decreased, all in a quadratic manner (Fig.8C). Later in the growing season (GDD≥0.5), *C*_i_ in Carbondale, was lower than at all other sites (*P* < 0.001), and *C*_i_ in Colby was greater than at the Manhattan and Carbondale sites (*P* ≤ 0.011). No differences in *C*_i_ among ecotypes were detected at any of the sites throughout the growing season (*P* ≥ 0.387).

#### Transpiration (*E*)

Leaf-level transpiration (*E*, mmol H_2_O m^−2^ s^−1^) showed patterns similar to those reported for other gas exchange measures, where a site-specific quadratic pattern was apparent for *E* over the growing season, regardless of ecotype (*P* = 0.002). For all ecotypes, differences in *E* between sites varied dynamically through the growing season. Early in the growing season (GDD = 0.4), Hays had greater *E* compared to the Manhattan and Carbondale planting sites (*P* < 0.02). From the middle of the growing season through the end of measures (GDD ≥ 0.5), the Hays site had significantly greater *E* compared to all other sites (*P* < 0.001), which were not significantly different from each other (*P* > 0.32).

Pairwise comparisons indicated differences in *E* mmol H_2_O m^−2^ s^−1^ between ecotypes at the drier sites in the early growing season. More specifically, at 0.3 GDD, the CKS ecotype had marginally greater *E* (6.2 ± 0.45, 6.5 ± 0.45) than the SIL ecotype (5.1 ± 0.45, 5.3 ± 0.45), in both Hays and Colby sites (*P* < 0.06). Also at the Hays site, the CKS ecotype had greater *E* than the EKS ecotypes (5.0 ± 0.45, *P* = 0.04). For most of the remaining growing season, there was no evidence for ecotypes differences at any of the sites. Nevertheless, at 0.8 GDD in Manhattan, KS, the CKS ecotype (4.8 ± 0.46) outperformed the SIL ecotype (3.2 ± 0.46) and showed greater *E* than the SIL ecotype (*P* < 0.01).

#### Intrinsic WUE

Intrinsic WUE, calculated as *A*/*g*_s_, had a site-specific quadratic pattern over the growing season (Fig.8E; *P* < 0.0001). Early in the growing season (GDD = 0.4), WUE in Hays and Carbondale was greater than in Manhattan (*P* ≤ 0.006), but none was significantly different from the Colby planting site, which showed an intermediate WUE. As the growing season progressed, the estimated WUE decreased quadratically in the drier sites [i.e. Hays and Colby (KS), Fig.8E], whereas WUE increased quadratically in the wettest site (i.e., Carbondale, IL). At the Manhattan planting site, WUE first increased and then decreased over the growing season, also following a quadratic pattern. By the end of the growing season (GDD = 0.7), all sites were significantly different from each other in their WUE and were ranked as Carbondale > Manhattan > Hays > Colby (*P* ≤ 0.016), that is, with higher WUE at wetter sites. Ecotype differences in WUE were apparent only at the Hays site early in the growing season (0.3 GDD), whereby the EKS ecotype (119 ± 3.7) had significantly greater WUE compared to the CKS (104.5 ± 3.7) and SIL (106 ± 3.7) ecotypes (*P* ≤ 0.015, *P* ≤ 0.033, respectively).

## Discussion

In this study, we document ecotypic variation in the dominant prairie grass *A. gerardii* across the climatic gradient of the US Great Plains. Within this variation, we found evidence of local adaptation in *A. gerardii* performance (establishment and vegetative cover) at xeric locations along the climatic gradient, suggesting prevailing local adaptation under abiotic stress. This suggests that in spite of gene flow, large population sizes, and an outbreeding mating system, we find clear evidence of local adaptation in some ecotypes. Thus, historical and current climate selection pressures appear strong enough to overcome homogenizing effects of gene flow (Gray et al. 2014) within this foundational species, as has been observed in other settings (Gonzalo-Turpin and Hazard 2009).

### Adaptive variation in *Andropogon gerardii* ecotypes

Local adaptation is defined as an interaction between ecotype and site (E × S), with ecotypes from local populations outperforming nonlocal transplants in subenvironments such as different climates (Linhart and Grant 1996). Furthermore, it is a fundamentally important mechanism in evolutionary, conservation, and global climate change biology, with applications for restoration (Hufford and Mazer 2003) and climate adaptation and mitigation (Jump and Penuelas 2005; Nicotra et al. 2010; Shaw and Etterson 2012). Plant populations commonly show local adaptation across scales or even within populations (Waser and Price 1985; Hangelbroek et al. 2003; Knight and Miller 2004; Lenssen et al. 2004). It is possible to predict local adaptation if divergent selection acts on phenotypes across a sharp environmental gradient, provided gene flow is low relative to the strength of selection, and phenotypic plasticity is limited in each environment (Van Tienderen 1997; Kawecki and Ebert 2004).

*Andropogon gerardii* is a wide-ranging species showing substantial variation across the environmental gradient of the Great Plains. Based on our studies from the same populations used here, *A. gerardii* ecotypes vary anatomically (Olsen et al. 2013), physiologically (Caudle et al. 2014), genetically (Gray et al. 2014), and morphologically (Gibson et al. 2013; Mendola et al. in press; M. Galliart, unpublished data). Plants originating from drier (western) sites flower 3 weeks earlier than plants sourced from wetter (eastern) sites (M. Galliart, unpublished data), as a putative adaption to drought. In addition, other studies focusing on belowground mycorrhizal symbionts indicate that local adaptation of *A. gerardii* performance may be explained in part on their specific and local mycorrhizal symbionts (Schultz et al. 2001; Johnson et al. 2010). Andropogon ecotypes adapt to their local soil arbuscular mycorrhizal fungal communities such that plants grown in home soil and inoculated with home AM fungi produced more arbuscules (symbiotic exchange structures) in their roots than those grown in away combinations.

In our study, we characterized this phenotypic variation and further detected evidence for local ecotypic adaptation for the CKS ecotype in the form of the longest timescale measurements of establishment and cover. At a shorter timescale, subtle ecotype differences were also detected in leaf chlorophyll absorbance, where the CKS ecotype exhibited higher SPAD values across sites relative to the other ecotypes of *A. gerardii*. This suggests CKS ecotype has a genetic predisposition that is beneficial in dry environments, at least in the first 2 years of establishment. Long-term measures (cover) will detect if this same pattern persists over time or changes dynamically.

Additional insights can be gleamed about the extent to which big bluestem can extend its range into even drier environments. Using our Colby site as a surrogate for harsher abiotic conditions, we can make predictions if there are certain ecotypes that might survive and maintain populations in the arid part of the Great Plains or under future drier climates. Although there were few differences in short-term measures of gas exchange, meaningful ecotypic differences were detected for the long-term measures of establishment and cover. In Colby, the CKS ecotype far outperformed (by a factor of 2–3 times) the SIL and EKS ecotypes in terms of establishment and cover. Interestingly, when planted with competition in Manhattan and Carbondale, there was no difference in establishment or cover between the CKS ecotype and the SIL and EKS counterparts, thereby suggesting local adaptation of the CKS ecotype to the more xeric region of the Great Plains. Moreover, greater success in vegetative performance of the CKS ecotype in Hays and Colby occurred despite no evidence for ecotypic differences in short-term gas exchange measures. This suggests the range of the CKS ecotype could potentially expand eastward with increased predicted drought.

Ecotype-specific responses to the environment also further highlight the differences among ecotypes. For instance, the functional form of the relationship between cover and historic precipitation (Fig.5) across the climatic gradient was specific to each ecotype, with a cubic shape for the CKS ecotype contrasted with a quadratic shape for the EKS and SIL ecotypes of *A. gerardii*. This suggests a role for climatic selection of ecotypes and their adaptive variation to abiotic stress tolerance at the extreme of the climatic range. Similar results on the role of adaptive variation to extreme ranges have also been found by Barnes (1985), Álvarez et al. (2012), and Beierkuhnlein et al. (2011).

Surprisingly, under favorable environmental conditions of increased rainfall (i.e. Manhattan and Carbondale planting sites), neither the EKS nor the SIL ecotypes showed any evidence for ‘home site advantage’. In contrast, both SIL and EKS ecotypes showed a quadratic functional relationship between cover and precipitation (Fig.5), such that the highest cover for these ecotypes occurred at intermediate rainfall, at least within the first 2 years of planting. Interestingly and contrary to prediction, in Carbondale, the site with the greatest rainfall and where we expected the highest cover and best performance of the SIL ecotype, all ecotypes showed the poorest establishment and cover of all sites in the first 2 years. We speculate that biotic interactions, at least initially, might exert greater pressure there than abiotic factors (Bischoff et al. 2006; Ariza and Tielborger 2011) as potential driving forces of local adaptation, particularly in conditions of low abiotic stress. More studies are needed to assess the abiotic and biotic factors as drivers of local adaptation (Bischoff et al. 2006) across the climatic gradient over time. Longer time to evaluate will allow us to determine whether this pattern holds beyond 2 years.

In contrast to the strong evidence for ecotypic differences on establishment, cover, and SPAD, planting site factors (not ecotypes) appeared to control gas exchange almost exclusively. Few ecotype differences in gas exchange were detected throughout the growing season, and any differences were of small magnitude. Instead, any anticipated home site advantage of the CKS ecotype was overshadowed by strong site effects on various components of short-term water use and gas exchange (Fig.8, Table 4). Gas exchange rates were highest early in the growing season and at the wettest sites. Furthermore, site differences in gas exchange did not mirror those found for cover or seedling measures. We highlight the different scale of measurement of these responses of interest, ranging from seconds (a typical period for gas exchange measurements) to weeks (a typical period for leaf and pigment development) to months and years (for establishment and cover measurements). Our results were somewhat surprising given the ecotype differences of photosynthesis and WUE found in other species (Knight et al. 2006; Flood et al. 2011). However, instantaneous measures of leaf-level gas exchange often fail to mirror the behavior of cover or biomass (Givnish 1988; Chiariello et al. 1989; Villar et al. 1998) and are quite sensitive to transient and local moisture, temperature, and light conditions (Field et al. 1989), masking ecotype differences. However, when ecotypes are grown without competition in monoculture, single-spaced plantings (not polyculture as reported here), the CKS ecotype indeed had the highest performance in gas exchange and SPAD (Caudle et al. 2014), similar to results in cover reported here from polyculture seeded plots.

One advantage of our study is we carried out our reciprocal garden experiment in a plant community (polyculture) with focal big bluestem and nonfocal species all planted in the field as seed, thus allowing us to test for local adaptation to biotic stress under more realistic ecological conditions. In contrast, many reciprocal garden experiments are carried out with weeded or herbicided, spaced plants in monoculture (McMillan 1959). Thus, our reciprocally seeded plots allowed us to account for selection and filtering at the critical stages of germination and establishment in a natural setting (Donohue et al. 2010). Seedlings grown under standardized greenhouse conditions and then planted in the field (Bischoff et al. 2006) may ignore the potential for adaptive population differentiation in seed survival, dormancy, and germination phases (Nagy and Rice 1997; Keller and Kollmann 1999; Galloway and Fenster 2000; Bischoff et al. 2006).

### Implications for restoration with changing climates

Our study has relevance for other grasslands worldwide, as grasslands are likely to experience increasing water stress (www/aqueduct.wri.org/atlas). Investigation of ecotypic variation of an ecologically dominant grass in the current and changing climate of the Great Plains is essential to make predictions about grassland response to climate change. The climate system of the Great Plains is dynamic, with increasing frequency and severity of current and predicted droughts (Weltzin et al. 2003; Knapp et al. 2008; IPCC 2013). Given the superior performance of the CKS ecotype under dry conditions and predicted continuation of drought in the Great Plains, we expect the CKS ecotype to migrate eastward, naturally or with assistance. However, it is unclear if this migration eastward might be hampered by strong differences in flowering times with the CKS ecotype flowering and senescing 3 weeks earlier than the SIL ecotype, no matter where it is planted on the gradient (M. Galliart, unpublished data).

Our study also helps to shape land management and conservation policies. Of particular interest, it lays the scientific foundation for land and conservation managers, such as which ecotypes to plant (Montalvo et al. 1997; Hufford and Mazer 2003; Falk et al. 2006; Harris et al. 2006; McKay et al. 2008) and how these ecotypes might perform in a competitive environment, as opposed to monocultures. The USDA Conservation Reserve program consists of ∼4.3 million acres in a five state Midwest region with the purpose to restore grasslands from marginal agricultural lands (SCS 1990) (http://www.fsa.usda.gov/programs-and-services/conservation-programs/reports-and-statistics/conservation-reserve-program-statistics/index). Indeed, *A. gerardii* is a foundation species used in these conservation plantings on marginal lands throughout the US grasslands. Furthermore, only 4% of historical tallgrass prairie remained in the United States. In the eastern Great Plains, where prairie conversion to row crop agriculture has been most intense, mere hundreds of hectares remain (Samson and Knopf 1994). Thus, restoring grasslands that are robust with respect to productivity, and drought tolerance is needed to optimize adaptive management strategies in US grasslands and worldwide, to ultimately provide for agricultural sustainability in the face of changing climates (Rice and Emery 2003; Harris et al. 2006).

Our study provides evidence for different *A. gerardii* ecotypes across precipitation zones with varying longitude, at the same latitude. Our ecotypes roughly correspond to the ecoregions (Bailey 1998) of Great Plains Steppe (CKS) and Great Plains Steppe/Prairie Parkland Temperate (EKS) and Prairie Parkland Temperate (southern IL) and analogous Plant Adaptation Regions identified by Vogel et al. (2005). Early switchgrass (*Panicum virgatum*) ecotype work of Casler et al. (2004, 2007a,b) suggests that plants sourced from varying longitude (in our case, varying precipitation) can be used for restoration provided they are derived from the same hardiness zone (within the same latitude). However, later studies (Casler 2012) recognize two east–west switchgrass germplasm groups, sorting by drought tolerance, a Prairie parkland ecotype (eastern) and Great Plains Steppe (western). This is similar to results reported here, recognizing a xeric *A. gerardii* ecotype, and cautiously emphasize planting across precipitation zones.

The potential to use local ecotypes for restoration practices (Hufford and Mazer 2003) is often recommended and might be warranted for *A. gerardii*, given the high within-population genetic diversity reported in these populations (Price et al. 2012; Gray et al. 2014). However, planting mixed population stands might provide the greatest buffer against future climate change (Lesica and Allendorf 1999; Jump and Penuelas 2005; Nicotra et al. 2010). However, the use of nonlocal varieties in restoration might have its own set of disadvantages, including reduced success and exchange of maladapted genes to local ecotypes through gene flow (Hufford and Mazer 2003; McKay et al. 2005; Edmands 2007). Given our results of the superior performance of the most xeric ecotype under dry conditions and predicted drought, this ecotype may migrate eastward, naturally or with assistance in restorations.

## Data archiving statement

Data available from the Dryad Digital Repository: http://dx.doi.org/10.5061/dryad.26fj6.

## Supporting Information

Table S1. Statistical analyses of establishment, cover, and SPAD chlorophyll absorbance. **Table S2.** Statistical analyses of photosynthesis, conductance, internal CO_2_, transpiration, and WUE.
