# Supplementary material for: Intraspecific variation of a dominant grass and local adaptation in reciprocal garden communities along a US Great Plains’ precipitation gradient: implications for grassland restoration with climate change
Source: Evol Appl. 2015 Jul 16;8(7):705–23. doi: 10.1111/eva.12281 (PMC4516422; doi:10.1111/eva.12281)
Supplement: Table S1. [file eva0008-0705-sd1.pdf]

Supplemental table 1. Statistical analyses of establishment, cover, and SPAD chlorophyll absorbance. Factors include planting site (S), Ecotype (E), for cover and SPAD also includes Growing Degree Day (GDD, both linear and quadratic) and all possible interactions.

FACTOR	ESTABLISHMENT		COVER		SPAD	
	F, $_{Df\,n,\,Df\,d}$	P>F	F, $_{Df\,n,\,Df\,d}$	P>F	F, $_{Df\,n,\,Df\,d}$	P>F
S	25.46, $_{3,\,10.42}$	**<0.0001**	7.93, $_{3,\,30.92}$	**0.0005**	6.99, $_{3,\,12}$	**0.0056**
E	8.73, $_{2,\,21.94}$	**0.0016**	0.76, $_{2,\,59.45}$	0.4716	0.46, $_{2,\,24}$	0.6395
E*S	0.46, $_{6,\,19.59}$	0.8322	4.33, $_{6,\,58.56}$	**0.0011**	0.56, $_{6,\,24}$	0.7609
GDD			0.05, $_{1,\,36}$	0.8287	37.66, $_{1,\,18}$	**<0.0001**
GDD*S			5.39, $_{3,\,36}$	**0.0036**	11.59, $_{3,\,118}$	**<0.0001**
GDD*E			4.68, $_{2,\,36.01}$	**0.0156**	0.50, $_{2,\,118}$	0.6096
GDD*S*E			1.17, $_{6,\,36.01}$	0.3424	0.66, $_{6,\,118}$	0.6846
GDD2					60.08, $_{1,\,118}$	**<0.0001**
GDD2*S					11.42, $_{3,\,118}$	**<0.0001**
GDD2*E					0.62, $_{2,\,118}$	0.5389
GDD2*S*E					0.76, $_{6,\,118}$	0.6016

Supplemental-Table 2. Statistical analyses of photosynthesis, conductance, internal CO_2, transpiration, and WUE. Factors include planting site (S), Ecotype (E), Growing Degree Day (GDD, both linear and quadratic) and all possible interactions.

FACTOR	PHOTOSYNTHESIS		CONDUCTANCE		INTERNAL CO_2		TRANSPIRATION		WUE	
	$F_{Df\,n,\,Df\,d}$	P	$F_{Df\,n,\,Df\,d}$	P	$F_{Df\,n,\,Df\,d}$	P	$F_{Df\,n,\,Df\,d}$	P	$F_{Df\,n,\,Df\,d}$	P
S	$5.68_{3,\,12}$	**0.011**	$18.91_{3,\,75.99}$	**<0.0001**	$7.54_{3,\,42.45}$	**0.0004**	$10.55_{3,\,104.2}$	**<0.0001**	$26.37_{3,\,42.01}$	**<0.0001**
E	$0.36_{2,\,24}$	0.698	$0.91_{2,\,75.14}$	0.40	$1.08_{2,\,60.49}$	0.3467	$2.32_{2,\,100.3}$	0.1039	$1.70_{2,\,56.48}$	0.19
E*S	$1.47_{6,\,24}$	0.229	$1.72_{6,\,75.46}$	0.1290	$0.27_{6,\,49.1}$	0.9487	$0.58_{6,\,99.64}$	0.74	$0.38_{6,\,47.37}$	0.88
GDD	$19.42_{1,\,72}$	**<0.0001**	$118.6_{1,\,72.84}$	**<0.0001**	$33.60_{1,\,57.64}$	**<0.0001**	$19.97_{1,\,96.41}$	**<0.0001**	$71.11_{1,\,60.55}$	**<0.0001**
GDD*S	$4.06_{3,\,72}$	**0.010**	$10.27_{3,\,72.62}$	**<0.0001**	$6.57_{3,\,43.16}$	**0.0009**	$9.65_{3,\,96.25}$	**<0.0001**	$19.67_{3,\,42.51}$	**<0.0001**
GDD*E	$0.63_{2,\,72}$	0.533	$0.64_{2,\,73.13}$	0.53	$0.96_{2,\,57.64}$	0.3874	$1.51_{2,\,98.89}$	0.22	$1.53_{2,\,59.7}$	0.22
GDD*S*E	$1.19_{6,\,72}$	0.319	$1.28_{6,\,72.83}$	0.27	$0.29_{6,\,49.88}$	0.9396	$0.49_{6,\,98.07}$	0.81	$0.47_{6,\,48.94}$	0.82
GDD2	$10.51_{1,\,72}$	**0.0018**	$98.33_{1,\,72.79}$	**<0.0001**	$35.76_{1,\,49.48}$	**<0.0001**	$14.55_{1,\,96.37}$	**0.0002**	$67.43_{1,\,55.21}$	**<0.0001**
GDD2*S	$4.44_{3,\,72}$	**0.0064**	$5.99_{3,\,72.65}$	**0.0011**	$3.51_{3,\,44.02}$	**0.0229**	$5.51_{3,\,96.27}$	**0.0016**	$9.49_{3,\,43.88}$	**<0.0001**
GDD2*E	$0.73_{2,\,72}$	0.48	$0.59_{2,\,73.06}$	0.5577	$0.82_{2,\,49.48}$	0.4447	$1.17_{2,\,98.69}$	0.31	$1.31_{2,\,54.75}$	0.27
GDD2*S*E	$0.98_{6,\,72}$	0.44	$1.00_{6,\,72.87}$	0.4294	$0.26_{6,\,50.86}$	0.9525	$0.53_{6,\,98.17}$	0.78	$0.43_{6,\,50.5}$	0.85
